# State-Level Guidance and District-Level Policies and Practices for Food Marketing in US School Districts

**DOI:** 10.5888/pcd15.170352

**Published:** 2018-06-07

**Authors:** Caitlin L. Merlo, Shannon Michael, Nancy D. Brener, Heidi Blanck

**Affiliations:** 1 Division of Population Health, Centers for Disease Control and Prevention, Atlanta, Georgia; 2 Division of Adolescent and School Health, Centers for Disease Control and Prevention, Atlanta, Georgia; 3 Division of Nutrition, Physical Activity, and Obesity, Centers for Disease Control and Prevention, Atlanta, Georgia

## Abstract

State agencies play a critical role in providing school districts with guidance and technical assistance on school nutrition issues, including food and beverage marketing practices. We examined associations between state-level guidance and the policies and practices in school districts regarding food and beverage marketing and promotion. State policy guidance was positively associated with districts prohibiting advertisements for junk food or fast food restaurants on school property. Technical assistance from states was negatively associated with 2 district practices to restrict marketing of unhealthy foods and beverages, but positively associated with 1 practice to promote healthy options. These findings may help inform the guidance that states provide to school districts and help identify which districts may need additional assistance to address marketing and promotion practices.

## Objective

State agencies (eg, state departments of education or health) play an important role in providing school districts with guidance and trainings related to federal meal program requirements. While the prevalence of food and beverage marketing practices in states, school districts, and schools in the United States has been described previously (1–4), it is not known whether the guidance that state agencies provide to school districts about marketing and promotion is associated with implementation of related policies and practices at the district level. Findings from our study could help inform state agencies’ assistance to school districts, especially in light of new federal rules requiring school districts to have local school wellness policies that address 1) food and beverage marketing and 2) nutrition promotion (5).

## Methods

We analyzed data from the 2012 School Health Policies and Practices Study (SHPPS). SHPPS is a nationally representative study periodically conducted by the Centers for Disease Control and Prevention to assess school health policies and practices at the state, district, school, and classroom levels. A detailed description of SHPPS, including the 2012 methodology, is available at https://www.cdc.gov/healthyyouth/shpps/index.htm (4,6).

Our analysis included questions from 3 SHPPS 2012 questionnaires: the state-level Nutrition Services questionnaire (response rate 100%; n = 51), the district-level Nutrition Services questionnaire (response rate 63.0%; n = 660 districts), and the district-level General School Environment questionnaire (response rate 60.1%; n = 630 districts). State and district data were linked to create a merged data set in which each district record contained variables about the guidance provided in that district’s state. All questions used in our analysis are shown in the Appendix.

We created 5 composite variables from the state-level questions. Three variables addressed states’ guidance to districts to restrict the marketing of unhealthy foods and beverages (developed or revised model policies [constructed from question 1f, 1g, and 1i; Cronbach α = 0.709], distributed or provided policy guidance [question 2f, 2g, and 2i; α = 0.709], and provided technical assistance [question 3f, 3g, and 3i; α = 0.669]). Two variables addressed states’ guidance to promote healthy foods and beverages: (provided technical assistance [question 3u and 3v] and provided professional development [question 6h, 6i, and 6j; α = 0.789]).

Four logistic regression models examined associations between state-level guidance (independent variables) and district policies and practices to restrict marketing of unhealthy foods and beverages (dependent variables). Two other models examined associations between state-level guidance and district policies and practices to promote healthy foods and beverages. All independent variables were included simultaneously in each of these models.

All models controlled for the following covariates: percentage of Title 1 students in the district, percentage of white students in the district, district size, district metro status, and total expenditures per student in the district. All analyses were conducted on weighted data using SUDAAN version 11.0.0 (RTI International) to account for the complex sample design.

## Results

Table 1 describes the frequency of state and district practices included in the models as well as the covariates studied. Findings for restricting marketing of unhealthy foods and beverages are in Table 2. The odds of a district requiring or recommending that schools prohibit advertisements for junk food or fast food restaurants on school property were 54% higher (AOR, 1.54; 95% CI, 1.14­–2.07) among districts in states that distributed or provided model policies, policy guidance, or other materials to district or school staff compared with districts that did not receive this assistance (Table 2). However, the odds of a district requiring or recommending that schools prohibit advertisements for junk food or fast food restaurants on school property were 41% lower (AOR, 0.59; 95% CI, 0.46–0.76) among districts in states that provided technical assistance to district or school staff compared with districts in states that did not. Additionally, the odds of a district requiring or recommending that schools restrict the distribution of products promoting junk food, fast food restaurants, or soft drinks to students were 29% lower (AOR, 0.71; 95% CI, 0.55–0.93) among districts in states that provided technical assistance relative to districts in states that did not. Compared with large districts, small districts had 70% lower odds of requiring or recommending that schools prohibit advertisements for junk food or fast food restaurants on school property (AOR, 0.30; 95% CI, 0.10–0.89), and 72% lower odds of requiring or recommending that schools prohibit junk foods from being sold for fundraising purposes (AOR, 0.28; 95% CI, 0.11–0.69).

**Table 1:** Descriptive Statistics for State and District Variables to Restrict Marketing of Unhealthy Foods and Beverages and to Promote Healthy Foods and Beverages — School Health Policies and Practices Study, 2012

Practice or Category	Value
**State practices (no. districts providing specific guidance), mean scale score (minimum–maximum)**	
State developed or revised policy on strategies to restrict marketing of unhealthy items^a^ (n = 706)	4.7 (3.0–6.0)
State distributed or provided policy guidance on strategies to restrict marketing of unhealthy items^b^ (n = 676)	4.7 (3.0–6.0)
State provided technical assistance on strategies to restrict marketing of unhealthy items^c^ (n = 706)	5.0 (3.0–6.0)
State provided funding for or offered professional development on strategies to increase participation in school meals^d^ (n = 742)	5.6 (3.0–6.0)
**State practices (no. districts providing specific guidance), % (95% confidence interval)**	
State developed or revised policy guidance on actively promoting fruits and vegetables, whole grain foods, and low-fat or nonfat dairy products to students (n = 684)	91.2 (87.7–93.8)
State distributed or provided policy guidance on actively promoting fruits and vegetables, whole grain foods, and low-fat or nonfat dairy products to students (n = 726)	96.7 (94.0–98.2)
State provided technical assistance to districts on marketing school meals and improving the presentation of healthy foods in the cafeteria^e^ (n = 690)	95.9 (93.4–97.5)
**District practices (sample size), % (95% confidence interval)**	
District requires or recommends that schools prohibit advertisement for junk food or fast food restaurants on school property^f^ (n = 399)	65.9 (61.8–69.8)
District requires or recommends that schools restrict the distribution of products promoting junk food, fast food restaurants, or soft drinks to students, such as T-shirts, hats, or book covers^f^ (n = 347)	57.1 (53.0–61.2)
District does not allow soft drink companies to advertise soft drinks in school buildings and/or other areas of school campus^g^ (n = 415)	79.5 (75.2–83.2)
District requires or recommends that schools prohibit junk foods from being sold for fundraising purposes (n = 372)^f^	58.4 (54.1–62.5)
District provided nutrition information to schools, students, and families^h^ (n = 310)	48.9 (44.6–53.1)
District provided professional development for nutrition services staff^i^ (n = 480)	77.4 (73.7–80.7)
**District characteristics (sample size), % (95% confidence interval)**	
**Percentage of Title 1 students in the district^j^**	
≤33 (n = 278)	39.2 (35.2–43.4)
>33 to <67 (n = 344)	47.1 (42.9–51.4)
≥67 (n = 108)	13.6 (11.0–16.8)
**Percentage of white students in the district**	
≤50 (n = 144)	16.1 (13.2–19.6)
>50 (n = 594)	83.9 (80.4–86.8)
**District size^k^**	
Small (n = 478)	67.8 (63.9–71.2)
Medium (n = 193)	25.8 (22.5–29.3)
Large (n = 73)	6.7 (5.1–8.4)
**District metro status^l^**	
Rural (n = 318)	47.7 (43.7–51.7)
Suburban (n = 113)	14.6 (11.7–18.0)
Urban (n = 310)	37.7 (33.9–41.7)
**Total expenditures per student in the district, $^m^**	
<8,850 (n = 368)	49.8 (44.8–54.8)
≥8,850 (n = 376)	50.2 (45.2–55.2)

a Composite variable combines responses to Q1f, Q1g, and Q1i regarding restriction of marketing of unhealthy foods and beverages. Scores were summed to calculate a scale score ranging from 3 to 6, with higher scores indicating more restricting practices. See Appendix for exact question wording.

b Composite variable combines responses to Q2f, Q2g, and Q2i regarding restriction of marketing of unhealthy foods and beverages. Scores were summed to calculate a scale score ranging from 3 to 6, with higher scores indicating more restricting practices. See Appendix for exact question wording.

c Composite variable combines responses to Q3f, Q3g, and Q3i regarding restriction of marketing of unhealthy foods and beverages. Scores were summed to calculate a scale score ranging from 3 to 6, with higher scores indicating more restricting practices. See Appendix for exact question wording.

d Composite variable combines responses to Q6h, Q6i, and Q6j regarding promotion of healthy foods and beverages. Scores were summed to calculate a scale score, ranging from 3 to 6, with higher scores indicating more promoting practices. See Appendix for exact question wording.

e Composite variable combines responses to Q3u and Q3v regarding promotion of healthy foods and beverages. Scores were summed and recoded to reflect a dichotomous response option of yes = 1 (provided technical assistance to districts on marketing school meals and improving the presentation of healthy foods in the cafeteria) or no = 2 (did not provide technical assistance on these topics.) See Appendix for exact question wording.

f For questions with response options require = 1, recommend = 2, or neither = 3, require and recommend responses were combined, and all responses were reverse coded so that neither = 0 and require/recommend = 1. See Appendix for exact question wording.

g Composite variable combines responses to Q130a and Q130b regarding restriction of marketing of unhealthy foods and beverages. Response options yes = 1 or no = 2 were summed and then recoded so that no ≥3 (ie, did not allow soft drink companies to advertise soft drinks in school buildings and/or other areas of school campus) and yes = 0 (ie, allow soft drink companies to advertise soft drinks in school buildings and/or other areas of school campus). See Appendix for exact question wording.

h Composite variables combines the following questions: Q17a, Q17b, Q18a, Q18b, and Q18c regarding promotion of healthy foods and beverages. Response options yes = 1 or no = 2 were summed so that no >5 (ie, did not provide information about school meals to students and families) and yes = 5 (ie, did provide information about school meals to students and families). See Appendix for exact question wording.

i Composite variable combines questions Q32d and Q32j regarding promotion of healthy foods and beverages. Response options yes = 1 or no = 2 were summed so that no >2 (ie, district did not provide funding for or offered professional development to nutrition services staff) and yes = 2 (ie, district provided funding for or offered professional development to nutrition services staff). See Appendix for exact question wording.

j Percentage of students receiving free or reduced-price lunch.

k Small = 1‒2,499 students; medium = 2,500‒9,999 students, and large ≥10,000 students.

l Metropolitan status defined as rural, suburban (large or small town), or urban (large central city, mid-sized central city, urban fringe of central city, urban fringe of mid-sized city).

m The median total annual expenditures per student (ie, instructional expenditures, support services, and noninstructional expenditures).

**Table 2:** Associations Between State and District Variables to Restrict Marketing of Unhealthy Foods and Beverages — School Health Policies and Practices Study, 2012^a^

Practice or Category	Model 1: District Requires or Recommends That Schools Prohibit Advertisement for Junk Food or Fast Food Restaurants on School Property^b^		Model 2: District Requires or Recommends That Schools Restrict the Distribution of Products Promoting Junk Food, Fast Food Restaurants, or Soft Drinks to Students (eg, T-shirts, Hats, Book Covers)^c^		Model 3: District Does Not Allow Soft Drink Companies to Advertise Soft Drinks in School Buildings and/or Other Areas of School Campus^d^		Model 4: District Requires or Recommends That Schools Prohibit Junk Foods From Being Sold for Fundraising Purposes^e^	
	AOR (95% CI)	*P* Value	AOR (95% CI)	*P* Value	AOR (95% CI)	*P* Value	AOR (95% CI)	*P* Value
State developed or revised policy on strategies to restrict marketing of unhealthy items	1.09 (0.80–1.50)	.58	1.23 (0.93–1.62)	.14	0.83 (0.58–1.20)	.32	1.20 (0.85–1.70)	.29
State distributed or provided policy guidance on strategies to restrict marketing of unhealthy items	1.54 (1.14–2.07)	<.01	1.17 (0.91–1.51)	.22	1.19 (0.81–1.74)	.37	1.04 (0.74–1.44)	.84
State provided technical assistance on strategies to restrict marketing of unhealthy items	0.59 (0.46–0.76)	<01	0.71 (0.55–0.93)	.01	0.93 (0.68–1.28)	.67	1.12 (0.86–1.45)	.40
**Percentage of Title 1 students in the district^f^**								
≤33	0.82 (0.38–1.78)	.62	0.94 (0.44–1.99)	.86	1.20 (0.54–2.64)	.65	0.84 (0.41–1.72)	.64
>33 to <67	0.91 (0.48–1.74)	.78	0.79 (0.41–1.52)	.48	1.35 (0.62–2.92)	.45	0.82 (0.44–1.55)	.55
≥67	1 [Reference]							
**Percentage of white students in the district**								
≤50	0.90 (0.49–1.65)	.74	0.77 (0.42–1.39)	.38	2.19 (0.92–5.24)	.08	1.11 (0.57–2.14)	.76
>50	1 [Reference]							
**District size^g^**								
Small	0.30 (0.10–0.89)	.03	0.38 (0.14–1.02)	.05	0.87 (0.22–3.44)	.84	0.28 (0.11–0.69)	.01
Medium	0.48 (0.16–1.44)	.19	0.37 (0.14–1.02)	.05	0.83 (0.21–3.28)	.79	0.53 (0.22–1.27)	.15
Large	1 [Reference]							
**District metro status^h^**								
Rural	1.16 (0.70–1.93)	.56	0.91 (0.54–1.52)	.71	0.72 (0.37–1.39)	.33	1.27 (0.77–2.08)	.34
Suburban	0.95 (0.51–1.78)	.88	0.77 (0.41–1.42)	.40	1.07 (0.47–2.42)	.88	1.10 (0.57–2.14)	.77
Urban	1 [Reference]							
**Total expenditures per student in the district, $^i^**								
<8,850	0.87 (0.57–1.35)	.54	1.19 (0.79–1.79)	.41	0.88 (0.48–1.60)	.66	1.24 (0.82–1.87)	.30
≥8,850	1 [Reference]							

Abbreviations: AOR, adjusted odds ratio; CI, confidence interval; SE, standard error.

a All models included the following covariates: percentage of Title 1 students in the district, percentage of white students enrolled in a district, district size, district metro status, and total expenditures per student. Sample sizes: model 1 n = 487, model 2 n = 480, model 3 n = 418, model 4 n = 510.

b Logistic regression with dependent variable Q121 and 3 independent variables: Q1f, Q1g, and Q1i; Q2f, Q2g, and Q2i; and Q3f, Q3g, and Q3i. Scores for each independent variable were summed to calculate a scale score ranging from 3 to 6, with higher scores indicating more restricting practices. See Appendix for exact question wording.

c Logistic regression with dependent variable: Q125 and 3 independent variables: Q1f, Q1g, and Q1i; Q2f, Q2g, and Q2i; and Q3f, Q3g, and Q3i. Scores for each independent variable were summed to calculate a scale score, ranging from 3 to 6, with higher scores indicating more restricting practices. See Appendix for exact question wording.

d Logistic regression with dependent variable: Q130a and Q130b and 3 independent variables: Q1f, Q1g, and Q1i; Q2f, Q2g, and Q2i; and Q3f, Q3g, and Q3i. Scores for each independent variable were summed to calculate a scale score, ranging from 3 to 6, with higher scores indicating more restricting practices. See Appendix for exact question wording.

e Logistic regression with dependent variable: Q52 and 3 independent variables: Q1f, Q1g, and Q1i; Q2f, Q2g, and Q2i; and Q3f, Q3g, and Q3i. Scores for each composite variable were summed to calculate a scale score, ranging from 3 to 6, with higher scores indicating more restricting practices. See Appendix for exact question wording.

f Percentage of students receiving free or reduced-price lunch.

g Small = 1‒2,499 students; medium = 2,500‒9,999 students, and large ≥10,000 students.

h Metropolitan status defined as rural, suburban (large or small town), or urban (large central city, mid-sized central city, urban fringe of central city, urban fringe of mid-sized city).

i The median total annual expenditures per student (ie, instructional expenditures, support services, and noninstructional expenditures).

Findings for promoting healthy foods and beverages are in Table 3. Districts in states that provided technical assistance on marketing school meals and improving the presentation of healthy foods in the cafeteria had 99% higher odds of providing funding for professional development to school nutrition staff on using the cafeteria for nutrition education and strategies to improve the presentation of healthful foods in the cafeteria compared with districts in states that did not receive this assistance (AOR, 1.99; 95% CI, 1.19–3.32) (Table 3). Compared with large districts, small districts had 70% lower odds of providing nutrition information (eg, nutrition information about the foods available to students) to schools, students, and families (AOR, 0.30; 95% CI, 0.13–0.70).

**Table 3:** Associations Between State and District Variables to Promote Healthy Foods and Beverages — School Health Policies and Practices Study, 2012^a^

Practice or Category	Model 1: District Provided Nutrition Information to Schools, Students, and Families^b^		Model 2: District Provided Professional Development for Nutrition Services Staff^c^	
	AOR (95% CI)	*P* Value	AOR (95% CI)	*P* Value
State developed or revised policy guidance on actively promoting fruits and vegetables, whole grain foods, and low-fat or nonfat dairy products to students	N/A		0.75 (0.28–2.03)	.57
State distributed or provided policy guidance on actively promoting fruits and vegetables, whole grain foods, and low-fat or nonfat dairy products to students	N/A		0.70 (0.18–2.74)	.61
State provided technical assistance to districts on marketing school meals and improving the presentation of healthy foods in the cafeteria	1.20 (0.35–4.16)	.77	1.99 (1.19–3.32)	.01
State provided funding for or offered professional development on strategies to increase participation in school meals	0.97 (0.74–1.28)	.85	0.88 (0.67–1.16)	.37
**Percentage of Title 1 students in the district^d^**				
≤33	0.97 (0.47–2.00)	.93	0.88 (0.37–2.06)	.77
>33 to <67	0.96 (0.49–1.90)	.91	0.98 (0.43–2.21)	.96
≥67	1 [Reference]			
**Percentage of white students in the district**				
≤50	1.00 (0.54–1.86)	.99	1.03 (0.53–2.02)	.92
>50	1 [Reference]			
**District size^e^**				
Small	0.30 (0.13–0.70)	.01	0.34 (0.11–1.05)	.06
Medium	0.47 (0.21–1.06)	.07	0.42 (0.14–1.26)	.12
Large	1 [Reference]			
**District metro status^f^**				
Rural	0.72 (0.46–1.13)	.15	1.04 (0.60–1.82)	.89
Suburban	0.70 (0.39–1.26)	.23	0.70 (0.37–1.32)	.27
Urban	1 [Reference]			
**Total expenditures per student in the district, $^g^**				
<8,850	1.25 (0.87–1.78)	.23	1.05 (0.69–1.61)	.82
≥8,850	1 [Reference]			

Abbreviations: AOR, adjusted odds ratio; CI, confidence interval.

a All models included the following covariates: percentage of Title 1 students in the district, percentage of white students enrolled in a district, district size, district metro status, and total expenditures per student. Sample sizes: model 1 n = 568 and model 2 n = 564.

b Logistic regression with dependent variable a composite variable of Q17a, Q17b, Q18a, Q18b, and Q18c. The 2 independent variables were 1) Q3u and Q3v and 2) Q6h, Q6i, and Q6j. Scores for Q6h, Q6i, and Q6j were summed to calculate a scale score, ranging from 3 to 6, with higher scores indicating more promoting practices. See Appendix for exact question wording.

c Logistic regression with dependent variable Q32d and Q32j. The 4 independent variables were 1) Q1h; 2) Q2h; 3) Q3u and Q3v; and 4) Q6h, Q6i, and Q6j. Scores for Q6h, Q6i, and Q6j were summed to calculate a scale score, ranging from 3 to 6, with higher scores indicating more promoting practices. See Appendix for exact question wording.

d Percentage of students receiving free or reduced-price lunch.

e Small = 1‒2,499 students; medium = 2,500‒9,999 students, and large ≥10,000 students.

f Metropolitan status defined as rural, suburban (large or small town), or urban (large central city, mid-sized central city, urban fringe of central city, urban fringe of mid-sized city).

g The median total annual expenditures per student (ie, instructional expenditures, support services, and noninstructional expenditures).

## Discussion

Findings showed a positive association between states providing policy guidance and districts restricting advertisements for junk food or fast food restaurants on school property. It is unclear why state provision of technical assistance was negatively associated with 2 of the practices to restrict marketing of unhealthy foods and beverages but positively associated with districts funding professional development on promoting healthy choices in the cafeteria. One possible explanation is that districts may be better equipped to provide professional development on promoting healthier options because several initiatives exist to support the work, including the US Department of Agriculture’s Team Nutrition training grants and resources (7) and the Smarter Lunchrooms Movement (8). However, fewer resources exist to help districts address marketing of unhealthy foods and beverages, and states potentially are focusing assistance efforts on districts that are not already restricting marketing of unhealthy foods and beverages. Our findings were similar to previous findings (1) that, compared with large districts, small districts had lower odds of implementing several key marketing and promotion policies and practices.

There are several limitations of our study. Because SHPPS is a cross-sectional study, causality between state assistance and district-level practices cannot be inferred. Other types of studies could be done to verify these associations, including longitudinal studies and natural experimental evaluations of changes in state guidance and in district practices. Additionally, SHPPS data are self-reported, and policies and practices were not verified using other sources. There also was poor alignment between some of the state- and district-level questions for the 2 models examining practices to promote healthy items. Future studies could examine these associations with state- and district-level data that more closely align as well as examine associations between state-level food and beverage marketing policies and school-level practices, because state policies have been important levers for other school nutrition changes (9,10). Future research could also try to identify other training and technical assistance topics that may help districts address food marketing, including identifying food and beverage marketing in the school setting and leveraging school wellness councils to address food marketing (1).

Despite inconsistent results, state agencies may be encouraged by findings that policy guidance and technical assistance to districts is associated with implementation of some marketing and promotion policies and practices. Smaller districts may need additional assistance to address marketing and promotion.

## Appendix State- and District-Level Questions Used in Analysis From the School Health Policies and Practices Study, 2012^a,b^

**Table:**

Policies and Practices	State-Level Questions (Independent Variables)	District-Level Questions^c^ (Dependent Variables)
**Restrict marketing of unhealthy foods and beverages**	**Q1**. During the past two years, did your state develop, revise, or assist in developing model policies, policy guidance, or other materials to inform district or school policy on each of the following topics? (Yes/No) **—f.** Discouraging the sale of less nutritious foods and beverages for school fund-raising campaigns **—g**. Discouraging the use of food or food coupons as a reward or punishment **—i.** Prohibiting advertising and promotion (e.g., signs, contests, and coupons) of less nutritious foods and beverages on school property **Q2.** During the past two years, did your state distribute or provide to district or school staff model policies, policy guidance, or other materials to inform district or school policy on each of the following topics? (Yes/No) **—f.** Discouraging the sale of less nutritious foods and beverages for school fund-raising campaigns **—g.** Discouraging the use of food or food coupons as a reward or punishment **—i.** Prohibiting advertising and promotion (e.g., signs, contests, and coupons) of less nutritious foods and beverages on school property **Q3.** During the past 12 months, has your state provided technical assistance to district or school staff on . . . (Yes/No) **—f.** Discouraging the sale of less nutritious foods or beverages for school fund-raising campaigns **—g.** Discouraging the use of food or food coupons as a reward or punishment **—i.** Prohibiting advertising and promotion (e.g., signs, contests, and coupons) of less nutritious foods and beverages on school property	**Q52.** Does your district require or recommend that schools prohibit junk foods from being sold for fundraising purposes? (Require, Recommend, Neither) **Q121.** Does your district require or recommend that schools prohibit advertisements for junk food or fast food restaurants on school property? (Require, Recommend, Neither) **Q125.** Does your district require or recommend that schools restrict the distribution of products promoting junk food, fast food restaurants, or soft drinks to students, such as T-shirts, hats, or book covers? (Require, Recommend, Neither) **Q130.^d^** Are soft drink companies allowed to advertise soft drinks, such as sports drinks, soda pop, or fruit drinks that are not 100% juice (Yes/No) **—a.** In school buildings? **—b.** What about on school grounds, including on the outside of school buildings, on playing fields, or other areas of campus?
**Promote healthy foods and beverages**	**Q1.** During the past two years, did your state develop, revise, or assist in developing model policies, policy guidance, or other materials to inform district or school policy on each of the following topics? (Yes/ No) **—h.** Actively promoting fruits and vegetables, whole grain foods, and low-fat or nonfat dairy products to students **Q2.** During the past two years, did your state distribute or provide to district or school staff model policies, policy guidance, or other materials to inform district or school policy on each of the following topics? (Yes/No) **—h.** Actively promoting fruits and vegetables, whole grain foods, and low-fat or nonfat dairy products to students **Q3**. During the past 12 months, has your state provided technical assistance to district or school staff on . . . (Yes/No) **—u.** Marketing healthful school meals? **—v.** Strategies to improve the presentation of healthful foods in the cafeteria? **Q6.** During the past two years, has your state provided funding for or offered professional development to nutrition services staff on . . . (Yes/No) **—h.** increasing the percentage of students participating in school meals? **—i.** Making school meals more appealing? **—j.** strategies to improve the presentation of healthful foods in the cafeteria?	**Q17.^e^** During the past 12 months, has anyone from your district . . . (Yes/No) **—a.** Made menus available to students? **—b.** Made information available to students on the nutrition and caloric content of foods available to them? **Q18**.^e^ During the past 12 months, has anyone from your district . . . (Yes/No) **—a.** Made menus available to families of all students? **—b.** Made information available to families of all students on the nutrition and caloric content of foods available to students? **—c.** Made information on the school nutrition services program available to families of all students? **Q32.^f^** During the past two years, has your district provided funding for or offered professional development to nutrition services staff on . . . (Yes/No) **—d.** Using the cafeteria for nutrition education? **—j.** Strategies to improve the presentation of healthful foods in the cafeteria?

^a^ Question numbering in the table reflects the numbering used in the School Health Policies and Practices Study questionnaires including state-level Nutrition Services questionnaire, district-level Nutrition Services questionnaire, and district-level General School Environment questionnaire available at https://www.cdc.gov/healthyyouth/shpps/questionnaires.htm.

^b^ Prevalence estimates for each variable included in this analysis are available at https://www.cdc.gov/healthyyouth/shpps/2012/pdf/shpps-results_2012.pdf#page=81.

^c^ For dependent variable questions with response options of require = 1, recommend = 2, or neither = 3. Require and recommend responses were combined, and all responses were reverse coded so that neither = 0 and require and recommend = 1.

^d^ For Q130a and Q130b, response options yes = 1 or no = 2 were summed and then recoded so that no ≥3 (ie, did not allow soft drink companies to advertise soft drinks in school buildings and/or other areas of school campus) and yes = 0 (ie, allow soft drink companies to advertise soft drinks in school buildings and/or other areas of school campus).

^e^ For Q17a, Q17b, Q18a, Q18b, and Q18c, response options yes = 1 or no = 2 were summed so that no >5 (ie, did not provide information about school meals to students and families) and yes = 5 (ie, did provide information about school meals to students and families).

^f^ For Q32d, and Q32j, response options yes = 1 or no = 2 were summed so that no >2 (ie, district did not provide funding for or offer professional development to nutrition services staff) and yes = 2 (ie, district provided funding for or offered professional development to nutrition services staff).
